# Predictors of conversion in laparoscopic Crohn’s surgery: a retrospective analysis of a tertiary cohort

**DOI:** 10.1007/s00464-026-13162-0

**Published:** 2026-07-20

**Authors:** Tommaso Violante, Stefano Cardelli, Giacomo Calini, Marco Novelli, Nicola Renzi, Lorenzo Gentilini, Nikolas Konstantine Dussias, Fernando Rizzello, Matteo Rottoli

**Affiliations:** 1 Surgery of the Alimentary Tract, IRCCS Azienda Ospedaliero-Universitaria Di Bologna, Bologna, Italy; 2 Department of Medical and Surgical Sciences, Alma Mater Studiorum - University of Bologna, Via Massarenti 9, 40138 Bologna, Italy; 3 Division of Colon and Rectal Surgery, Mayo Clinic, Rochester, MN USA; 4 Department of Statistical Sciences, Alma Mater Studiorum - University of Bologna, Bologna, Italy; 5 Inflammatory Bowel Disease (IBD) Unit, IRCCS Azienda Ospedaliero-Universitaria Di Bologna, University of Bologna, Bologna, Italy

**Keywords:** Crohn’s disease, Laparoscopic surgery, Conversion, Ileocecal resection, Penalized regression

## Abstract

**Background:**

Laparoscopy is the preferred approach for ileocecal resection in Crohn’s disease, yet conversion to open surgery remains clinically significant. This study aimed to identify independent preoperative predictors of conversion using penalized regression (LASSO) and evaluate its impact on postoperative safety.

**Methods:**

A retrospective cohort study included adult patients undergoing elective laparoscopic ileocecal resection at a tertiary center between 2011 and 2025. Multivariable logistic regression with least absolute shrinkage and selection operator (LASSO) regularization was performed to identify robust predictors of conversion while minimizing overfitting. Postoperative outcomes were compared between laparoscopic and converted groups.

**Results:**

Of 575 patients, 90 (15.7%) required conversion. LASSO analysis identified older age (OR 1.02; 95% CI 1.00–1.04; *p* = 0.024) and fistulizing behavior (OR 1.98; 95% CI 1.08–3.62; *p* = 0.026) as independent risk factors. Conversely, a stenotic phenotype was protective (OR 0.53; 95% CI 0.29–0.96; *p* = 0.036). While conversion was associated with longer hospital stays (*p* = 0.009) and increased medical complications (25.6% vs. 14.4%; *p* = 0.013), it did not increase the rate of major surgical complications (7.8% vs. 6.4%; *p* = 0.799) or reoperations (*p* = 1.000).

**Conclusion:**

Conversion was independently predicted by older age and fistulizing disease, whereas stenotic behavior was protective. Although associated with higher medical morbidity, conversion did not compromise surgical safety, supporting its role as a proactive maneuver for complex disease rather than a technical failure.

Crohn’s disease is a chronic, relapsing inflammatory bowel disease, and most patients ultimately require at least one surgical intervention during their lifetime [1]. Ileocecal resection is the most frequently performed operation and is recommended as the standard surgical treatment for localized terminal ileal disease when medical therapy fails or complications develop [2].

Over the past two decades, laparoscopy has become the preferred approach for ileocecal resection and is widely regarded as the gold-standard technique for suitable cases, as it provides reduced postoperative pain, shorter hospital stay, faster recovery, and superior cosmetic results compared with open surgery [3–6]. Despite these advantages, conversion from laparoscopic to open surgery remains a clinically relevant issue, with reported conversion rates in contemporary series generally ranging from approximately 10% to over 37% [7]. Conversion is not merely a change in access; it has been associated with longer hospitalization, and higher healthcare costs, underscoring the need for careful preoperative risk stratification [8, 9].

Several studies have identified potential predictors of conversion in Crohn’s disease, including obesity, previous abdominal surgery, and intra-abdominal abscesses or fistulas, although findings are not entirely consistent [10–12]. In particular, the impact of specific disease phenotypes, namely inflammatory, stricturing, and penetrating behavior, on conversion risk remains unclear, and complex disease is often presumed to mandate an open approach or early conversion, even though experienced high-volume centers may safely manage these patients laparoscopically with modern minimally invasive techniques [13].

Against this background, the present study aimed to identify independent preoperative predictors of conversion to open surgery in a large cohort of patients undergoing elective laparoscopic ileocecal resection for Crohn’s disease at a tertiary referral center. Specifically, we sought to clarify the influence of disease behavior on the likelihood of conversion and to determine whether conversion itself represents an independent risk factor for adverse postoperative outcomes.

## Methods

### Study design and patient selection

This retrospective cohort study included patients undergoing elective surgery for Crohn’s disease. After receiving Institutional Review Board approval from the Comitato Etico di Area Vasta Emilia Centro (Approval: 270/2024/Oss/AOUBo), data were obtained. The study population comprised all adult patients (≥ 18 years) who underwent ileocecal resection with a planned laparoscopic approach between May 2011 and October 2025. This study was prepared in accordance with the Strengthening the Reporting of Cohort Studies in Surgery (STROCSS) guideline. Patients were categorized into two groups according to the final surgical approach: a laparoscopic group, including procedures completed entirely laparoscopically, and a conversion group, including procedures requiring conversion to open surgery. Conversion to open surgery was defined as any unplanned laparotomy, or the early extension of a planned extraction incision, required to complete the intra-abdominal dissection, bowel mobilization, or vascular ligation due to technical difficulties or safety concerns. Furthermore, provided that the entirety of the intended minimally invasive dissection was successfully completed, the enlargement of an extraction incision strictly to accommodate the removal of a bulky, specimen was not classified as a conversion. Patients who underwent primary open surgery or procedures other than ileocecal resection were excluded from the analysis.

### Data collection and definitions

Preoperative variables included patient demographics (age, sex, body mass index [BMI], smoking status), clinical status (the American Society of Anesthesiologists [ASA] score, disease duration), and surgical history (previous abdominal surgery). Disease behavior was classified according to the Montreal classification as B1 (non-stricturing/non-penetrating), B2 (stricturing), or B3 (penetrating). Preoperative medical therapy (systemic steroids, biologic agents, immunomodulators) and the presence of preoperative complications (e.g., intra-abdominal abscess) were also recorded.

Intraoperative variables included operative time and the creation of a stoma. Postoperative outcomes were assessed within 30 days of surgery and included length of hospital stay (LOS), medical complications, surgical complications, need for reoperation, and mortality. Postoperative complications were graded according to the Clavien–Dindo classification; major complications were defined as grade III or higher, corresponding to events requiring surgical, endoscopic, or radiological intervention.

### Statistical analysis

Categorical variables are presented as frequencies and percentages, whereas continuous variables are reported as median with interquartile range (IQR). Univariate analyses were performed to compare baseline characteristics and postoperative outcomes between the laparoscopic and conversion groups. The Chi-square test or Fisher’s exact test was applied for categorical variables, and the Mann–Whitney *U* test was used for continuous variables.

To identify independent predictors of conversion to open surgery, a multivariable logistic regression model was developed. Variable selection was performed using the least absolute shrinkage and selection operator (LASSO) method. Unlike traditional stepwise selection, LASSO applies a penalization (L1 regularization) to regression coefficients, shrinking non-informative variables to zero to minimize overfitting and address multicollinearity among predictors. The optimal penalization parameter (lambda) was determined via tenfold cross-validation. Variables with non-zero coefficients in the LASSO model were retained for the final multivariable analysis. Results are reported as odds ratios (OR) with corresponding 95% confidence intervals (CI), and a two-sided *p* value < 0.05 was considered statistically significant. All analyses were performed using Stata SE version 18.0 (StataCorp, College Station, TX, USA). A qualified statistician (M.N.) was actively involved in the study design and supervision of statistical methodology.

## Results

### Patient selection and demographics

A total of 575 patients underwent elective ileocecal resection for Crohn’s disease with a planned laparoscopic approach. The procedure was successfully completed laparoscopically in 485 patients (84.3%), while 90 patients (15.7%) required conversion to open surgery. Baseline characteristics are compared in Table 1. Patients in the conversion group were significantly older, with a median age of 37.5 years compared to 34.0 years in the laparoscopic group (*p* = 0.045). The conversion group also had a significantly higher median BMI of 22.1 kg/m^2^ compared to 21.0 kg/m^2^ in the laparoscopic group (*p* = 0.040). No significant differences were found regarding sex, smoking status, ASA score, disease duration, or the use of preoperative medications including steroids and biologics.

**Table 1:** Patient characteristics

Characteristic	Converted (*n* = 90)	Laparoscopic (*n* = 485)	*p* value
Age (years), median (IQR)	37.5 (28.0–51.0)	34.0 (25.8–48.0)	**0.045**
Male sex	48 (53.3%)	236 (48.7%)	0.484
BMI (kg/m^2^), median (IQR)	22.1 (19.9–25.3)	21.0 (18.9–23.7)	**0.040**
Current smoker	11 (12.2%)	84 (17.3%)	0.298
ASA Score:			0.962
ASA 1	24 (26.7%)	136 (28.0%)	
ASA 2	58 (64.4%)	312 (64.3%)	
ASA 3	8 (8.9%)	37 (7.6%)	
Disease duration (years), median (IQR)	7.4 (2.5–13.5)	5.8 (2.4–11.7)	0.248
Previous abdominal surgery	25 (27.8%)	129 (26.6%)	0.918
Disease behavior:			** < 0.001**
Inflammatory (B1)	4 (4.4%)	23 (4.7%)	
Stenotic (B2)	51 (56.7%)	386 (79.6%)	
Fistulizing (B3)	52 (57.8%)	160 (33.0%)	
Perianal disease	6 (6.7%)	60 (12.4%)	0.168
Preoperative abscess	32 (35.6%)	82 (16.9%)	** < 0.001**
Preoperative steroids	24 (26.7%)	119 (24.5%)	0.767
Preoperative biologics	35 (38.9%)	190 (39.2%)	1.000
Preoperative immunomodulators	2 (2.2%)	27 (5.6%)	0.291

Bold values indicate statistical significance (*p* < 0.05)

Data are presented as median (IQR) for continuous variables and as number (percentage) for categorical variables. *p* values were calculated using the Mann–Whitney *U* test for continuous data, and the Chi-square or Fisher’s exact test for categorical data. *BMI* body mass index, *ASA* American Society of Anesthesiologists

### Disease phenotype

Disease behavior, classified by the Montreal Classification, was a major driver of the surgical approach. The conversion group exhibited a significantly higher prevalence of fistulizing disease (57.8% vs. 33.0%; *p* < 0.001) and preoperative intra-abdominal abscesses (35.6% vs. 16.9%; *p* < 0.001). In contrast, patients with stenotic disease were significantly more likely to undergo successful laparoscopic resection (79.6% vs. 56.7%; *p* < 0.001). The presence of perianal disease was similar between groups, occurring in 6.7% of converted cases and 12.4% of laparoscopic cases (*p* = 0.168).

### Surgical outcomes and safety

Perioperative outcomes are detailed in Table 2. Conversion was associated with a longer median operative time of 180.0 min compared to 165.0 min (*p* = 0.011) and a prolonged median length of hospital stay of 7.0 days, with a wider interquartile range in the converted group (*p* = 0.009). Regarding safety, medical complications occurred significantly more frequently in the conversion group (25.6% vs. 14.4%; *p* = 0.013). As shown in Table 3, this difference was primarily driven by a significantly higher rate of respiratory complications such as pneumonia or effusion (6.7% vs. 2.5%; *p* = 0.038). Overall surgical complication rates were comparable between the converted and laparoscopic groups (21.1% vs. 19.4%; *p* = 0.814), including the incidence of major complications (Clavien-Dindo ≥ III: 7.8% vs. 6.4%; *p* = 0.799). A targeted review of the most severe events confirmed that conversion did not precipitate high-grade morbidities, as life-threatening complications (CD IV) were entirely absent in the converted cohort, and the frequency of events requiring reintervention under general anesthesia (CD IIIb) remained proportionally similar to the laparoscopic group.

**Table 2:** Surgical outcomes

Outcome	Converted (*n* = 90)	Laparoscopic (*n* = 485)	*p *value
Operative time (min)	180.0 (145.0–235.0)	165.0 (139.0–210.0)	**0.011**
Length of stay (days)	7.0 (6.0–9.0)	7.0 (6.0–8.0)	**0.009**
Stoma created	6 (6.7%)	14 (2.9%)	0.138
Surgical complications	19 (21.1%)	94 (19.4%)	0.814
Medical complications	23 (25.6%)	70 (14.4%)	**0.013**
Major complication (CD ≥ 3)	7 (7.8%)	31 (6.4%)	0.799
Reoperation	4 (4.4%)	23 (4.7%)	1.000
Readmission (30 days)	7 (7.8%)	34 (7.0%)	0.785
Mortality	0 (0.0%)	2 (0.4%)	1.000

Bold values indicate statistical significance (*p* < 0.05)

Outcomes are compared between patients who underwent successful laparoscopic resection and those requiring conversion to open surgery. Data are presented as median (IQR) or *n* (%). *p* values were calculated using the Mann–Whitney *U* test for continuous data, and the Chi-square or Fisher’s exact test for categorical data Major complications are defined as Clavien–Dindo grade ≥ III. *CD* Clavien–Dindo classification

**Table 3:** Detailed breakdown of postoperative complications

Complication	Converted (*n* = 90)	Laparoscopic (*n* = 485)	*p* value
Surgical complications	**19 (21.1%)**	**94 (19.4%)**	**0.814**
Wound infection (SSI)	8 (8.9%)	22 (4.5%)	0.082
Intra-abdominal abscess	5 (5.6%)	18 (3.7%)	0.407
Anastomotic leak	3 (3.3%)	12 (2.5%)	0.672
Postoperative ileus	9 (10.0%)	29 (6.0%)	0.165
Postoperative bleeding	2 (2.2%)	6 (1.2%)	0.612
Stoma complication	1 (1.1%)	3 (0.6%)	0.548
Medical complications	**23 (25.6%)**	**70 (14.4%)**	**0.013**
Respiratory (pneumonia/effusion)	6 (6.7%)	12 (2.5%)	**0.038**
Cardiac (arrhythmia/ischemia)	4 (4.4%)	9 (1.9%)	0.138
Urinary tract infection (UTI)	3 (3.3%)	14 (2.9%)	0.692
Renal (AKI)	2 (2.2%)	4 (0.8%)	0.236
Thromboembolic (DVT/PE)	1 (1.1%)	3 (0.6%)	0.548
Sepsis (non-abdominal)	2 (2.2%)	5 (1.0%)	0.312
Other medical	5 (5.6%)	23 (4.7%)	0.765

Bold values indicate statistical significance (*p* < 0.05)

Data are presented as number (percentage). *p* values were calculated using the Chi-square test or Fisher’s exact test as appropriate. *SSI* surgical site infection, *CD* Clavien–Dindo Classification, *UTI* urinary tract infection, *AKI* acute kidney injury, *DVT* deep vein thrombosis, *PE* pulmonary embolism

The rate of surgical complications was comparable between groups (21.1 vs. 19.4%; *p* = 0.814). Analysis of specific surgical adverse events revealed no significant differences in the rates of wound infection (8.9 vs. 4.5%; *p* = 0.082), postoperative ileus (10.0 vs. 6.0%; *p* = 0.165), intra-abdominal abscess (5.6 vs. 3.7%), anastomotic leak (3.3 vs. 2.5%), or postoperative bleeding.

### Predictors of conversion

In the multivariable analysis using LASSO regularization to adjust for baseline confounders, disease phenotype, and age emerged as the strongest independent predictors of conversion (Table 4). Fistulizing disease behavior was associated with a nearly twofold increase in the risk of conversion (OR 1.98; 95% CI 1.08–3.62; *p* = 0.026). Conversely, stenotic behavior was identified as a protective factor, associated with a significantly lower likelihood of conversion compared to inflammatory disease (OR 0.53; 95% CI 0.29–0.96; *p* = 0.036).

**Table 4:** Predictors of conversion to open surgery (LASSO regression)

Variable	Importance (standardized coefficient)^*^	Adjusted OR (95% CI)	*p* value
Fistulizing behavior (B3)	** + 0.28**	**1.98** (1.08–3.62)	**0.026**
Stenotic behavior (B2)	**−0.23**	**0.53** (0.29–0.96)	**0.036**
Age (years)	** + 0.18**	**1.02** (1.00–1.04)	**0.024**
Preoperative abscess	+ 0.18	1.75 (0.97–3.15)	0.061
BMI (kg/m^2^)	+ 0.10	1.05 (0.99–1.11)	0.118
Perianal disease	−0.10	0.51 (0.21–1.26)	0.146
Current smoker	−0.08	0.60 (0.28–1.30)	0.196
Immunomodulators	−0.10	0.26 (0.03–2.02)	0.200
ASA score	−0.04	0.70 (0.38–1.28)	0.246

Bold values indicate statistical significance (*p* < 0.05)

Predictors identified by LASSO logistic regression with post-selection adjusted odds ratios

Odds ratios (OR) are presented with 95% confidence intervals (CI). Reference category for disease behavior is inflammatory (B1). *OR* odds ratio, *CI* Confidence interval, *BMI* body mass index, *ASA* American Society of Anesthesiologists

^*^Standardized coefficients from the LASSO model indicate relative variable importance. Positive values increase risk; negative values are protective

Older age was also found to be an independent risk factor, with the odds of conversion increasing by 2% for every additional year of age (OR 1.02; 95% CI 1.00–1.04; *p* = 0.024). Preoperative abscess was identified by the LASSO model as a high-importance variable (standardized coefficient + 0.18) and showed a strong trend toward increased risk (OR 1.75; 95% CI 0.97–3.15; *p* = 0.061), though it did not reach strict statistical significance. Factors such as BMI, previous abdominal surgery, sex, and preoperative medication use (steroids, biologics) were not independent predictors of conversion.

### Model performance and validation

**Fig. 1:**
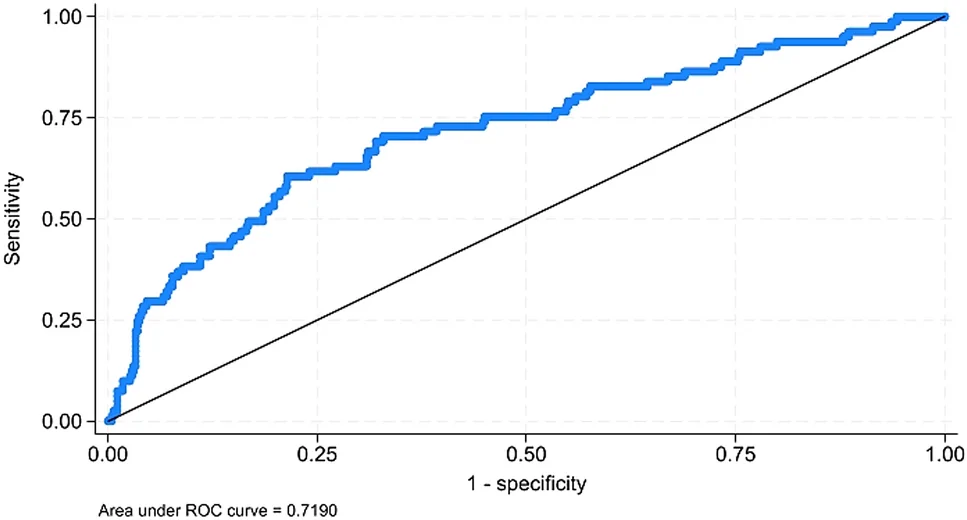
Receiver operating characteristic (ROC) curve for the prediction of conversion to open surgery. The curve illustrates the discriminative ability of the multivariable logistic regression model in predicting conversion from laparoscopic to open ileocecal resection. Predictors were selected using least absolute shrinkage and selection operator (LASSO) regularization to identify the most robust risk factors

The discriminative ability of the final multivariable model, incorporating the LASSO-selected predictors, was assessed using receiver operating characteristic (ROC) analysis. The model demonstrated fair to good predictive performance, yielding an area under the curve of 0.719 (Fig. 1).

## Discussion

In this large single-center cohort of elective laparoscopic ileocecal resections for Crohn’s disease, conversion to open surgery occurred in 15.7% of cases. Older age and fistulizing behavior independently increased the risk of conversion, whereas a stricturing phenotype was protective. The prediction model showed fair discrimination, and conversion was not independently associated with higher surgical complication or reoperation rates, although it was linked to longer operative time, slightly prolonged hospital stay, and more medical complications.

The 15.7% conversion rate observed in this cohort is consistent with published series, where conversion rates range from approximately 10 to 37% depending on patient selection and disease complexity [7]. In line with prior evidence, older age and fistulizing disease emerged as key predictors of conversion in our cohort, with dense adhesions frequently reported as the main intraoperative reason for early conversion [14]. Multiple studies have shown that complex Crohn’s disease is associated with a substantially higher likelihood of conversion; however, unlike earlier reports, previous abdominal surgery was not independently associated with conversion to open surgery in our analysis, in keeping with findings from a recent smaller study [15]. A recent article by Carvello et al. identified male sex, BMI, preoperative evidence of multiple disease localizations and abscess or perforation, and previous surgery for Crohn’s disease as independent risk factors for conversion [16]. In our experience, these results only partially overlapped with our practice’s outcomes, suggesting that discrepancies may reflect differences in case mix, institutional volume, and familiarity with minimally invasive management of complex Crohn’s disease.

While a stenotic (B2) phenotype was generally protective against conversion in our cohort, cases that did require conversion presented specific intraoperative challenges. A detailed review of the strictly B2 converted patients revealed that 91.1% were converted due to technical difficulty related to severe inflammation and altered anatomy (failure to progress), while only 8.9% were converted due to emergent intraoperative complications. Although our database does not record the exact timing of conversion (e.g., immediate upon diagnostic laparoscopy versus delayed after a trial of dissection), these findings highlight that the decision to convert in this subgroup is predominantly a proactive strategy driven by technical complexity rather than an emergent safety rescue.

A critical clinical observation from this study is that conversion did not compromise core surgical safety endpoints. While converted patients experienced longer hospital stays [7.0 (6.0–9.0) vs. 7.0 (6.0–8.0) days; *p* = 0.009] and higher rates of medical complications (25.6 vs. 14.4%; *p* = 0.013)—driven primarily by respiratory events—their rates of major surgical complications (Clavien–Dindo ≥ III: 7.8 vs. 6.4%; *p* = 0.799), reoperations (4.4 vs. 4.7%; *p* = 1.000), and 30-day readmissions (7.8 vs. 7.0%; *p* = 0.785) were nearly identical to those successfully managed laparoscopically. These reassuring findings, corroborated primarily by smaller series to date [17–19], reinforce the concept that conversion functions as an intraoperative safety maneuver rather than a technical failure, enabling surgeons to proactively escalate care when faced with unsafe dissection planes or obscured anatomy without incurring excess surgical morbidity [20–22]. Nevertheless, given the shorter length of stay in the laparoscopic group we continue to advocate an initial laparoscopic approach whenever feasible, with early conversion prompted by lack of progress rather than waiting for intraoperative complications to arise.

From a clinical standpoint, these findings support a more nuanced approach to both surgical planning and preoperative counseling. Rather than treating laparoscopy as a binary choice, the identified predictors allow surgeons to frame conversion as a probabilistic event that can be anticipated in specific phenotypes, particularly older patients with fistulizing disease [23, 24]. Discussing this risk explicitly, while emphasizing that conversion did not increase major surgical morbidity or reoperation in this and other cohorts, may help recalibrate patient expectations and reduce the perception of conversion as a “complication” or technical failure [25–27]. In practice, simple stratification into low-, intermediate-, and high-conversion risk profiles could be incorporated into shared decision-making and consent processes, informing not only patient counseling, but also the allocation of operative time, the choice of surgeon and team, and postoperative monitoring intensity, while still advocating an initial minimally invasive approach whenever feasible.

Several limitations of the present study warrant consideration. First, the retrospective, single-center design may restrict the generalizability of our findings, particularly to lower-volume surgical units that may lack extensive experience in complex minimally invasive surgery for inflammatory bowel disease. Second, while the multivariable model identified significant predictors, its discriminative ability, as indicated by an AUC of 0.71, remains moderate. This fair performance suggests that while the model is useful for preoperative counseling and resource allocation, it should not be utilized as a definitive tool to deny patients a laparoscopic attempt. Third, the model currently lacks external validation; it is essential that these results are tested in independent cohorts before the scoring system can be formally implemented for clinical decision-making or case triage. Furthermore, the increasing adoption of the robotic approach for complex Crohn’s disease may limit the generalizability of these findings, as the enhanced visualization and articulation of robotic platforms might alter the thresholds for conversion compared to standard laparoscopy. From a methodological standpoint, traditional multivariable logistic regression approaches, which include all known confounders and effect modifiers as moderators, can be limited by overfitting and multicollinearity, particularly when analyzing multiple interacting disease phenotypes and patient factors. In contrast, the LASSO method applies L1 regularization to shrink non-informative variables to zero, offering the advantage of a more parsimonious, robust model that highlights the most critical predictors of conversion. However, a limitation of this strategy is that it may penalize and exclude traditional confounders with smaller effect sizes that could still be clinically relevant in specific patient subgroups. Finally, as with any study utilizing a prospectively maintained database, there is an inherent risk of unmeasured confounding variables that could influence both the likelihood of conversion and the reported postoperative outcomes.

## Conclusion

Conversion to open surgery occurred in 15.7% of cases and was independently predicted by older age and fistulizing disease behavior, while a stenotic phenotype was protective. Although converted patients experienced longer hospital stays and more medical complications, conversion was not an independent risk factor for major surgical complications or reoperation. These findings suggest that while clinical phenotypes can help stratify risk, conversion should be viewed as a safe, proactive strategy for managing complex Crohn’s disease rather than a technical failure.
